# Extent of Groin Dissection in Melanoma: A Mixed-Methods, Population-Based Study of Practice Patterns and Outcomes

**DOI:** 10.3390/curroncol28060452

**Published:** 2021-12-16

**Authors:** Suzana Küpper, Janice L. Austin, Brittany Dingley, Yuan Xu, Kristine Kong, Mantaj Brar, Frances C. Wright, Carolyn Nessim, Antoine Bouchard-Fortier, May Lynn Quan

**Affiliations:** 1 University of Alberta, Edmonton, AB T6G 2R3, Canada; 2 University of Toronto, Toronto, ON M5S 1A1, Canada; 3 The Ottawa Hospital, University of Ottawa, Ottawa, ON K1H 8L6, Canada; 4 University of Calgary, Calgary, AB T2N 1N4, Canada

**Keywords:** melanoma, groin metastases, surgery

## Abstract

Melanoma metastases to the groin are frequently managed by therapeutic lymph node dissection. Evidence is lacking regarding the extent of dissection required. Thus, we sought to describe practice patterns for the use of inguinal vs. ilioinguinal dissection, as well as the perioperative/oncologic outcomes of each procedure. A mixed-methods approach was employed to evaluate surgical practice patterns. A retrospective review of three multi-site databases was carried out, together with semi-structured interviews of melanoma surgeons. A total of 347 patients who underwent dissection were reviewed. The main indications stated for adding a “deep” ilioinguinal dissection were palpable or radiologically positive disease. There was no significant difference in complications, length of stay or lymphedema between patients having inguinal vs. ilioinguinal dissection, irrespective of method of diagnosis. There was also no significant difference in recurrence, cancer-specific survival or overall survival between groups. In conclusion, ilioinguinal dissection is a safe and well-tolerated procedure, with no significant added morbidity relative to an inguinal dissection. The indications for ilioinguinal dissection currently in use produce an appropriate deep node positivity rate and ilioinguinal dissection should continue to be used selectively. Randomized data are needed to clarify the impact of ilioinguinal dissection on regional control and survival.

## 1. Introduction

Melanoma is a common malignancy with various subtypes including cutaneous, acral, mucosal and uveal. These different subtypes exhibit unique biological and clinical behaviour with significant resultant differences in prognosis [1]. Cutaneous melanoma is the most common subtype and the incidence is increasing worldwide, with an estimated 7800 new cases and 1300 melanoma-related deaths in Canada in 2019 [2]. Traditionally, wide local excision of the primary lesion and sentinel lymph node biopsy (SLNB) in clinically node negative patients is undertaken for curative intent, with those found to have sentinel lymph node metastases managed with therapeutic lymph node dissection. More recently, data from the DeCOG-SLT trial showed no difference in survival in patients treated with completion lymph node dissection vs. observation [3]. Similarly, the MSLT-II trial comparing completion lymph node dissection with observation in SLNB positive patients found greater prognostic value but no difference in melanoma specific survival [4]. There remains a subgroup of patients, however, for whom therapeutic dissection is indicated, such as those with clinically evident disease and those who develop disease during ultrasound surveillance after a positive SLNB. For metastases in the groin, the extent of dissection can include just the superficial lymph nodes (inguinal node dissection) or a combination of the superficial lymph nodes and the deep lymph nodes along the iliac and obturator vessels in the pelvis (ilioinguinal dissection).

To date, clear evidence is lacking regarding the extent of dissection required in the presence of inguinal disease. Specifically, data is lacking on whether the addition of an ilioinguinal dissection (i.e., a deep pelvic lymph node dissection) improves patient outcomes. Given that dissection of the groin carries increased complications and rates of lymphedema compared to other nodal basins, some surgeons are choosing to forgo the more extensive dissection entirely. There is an ongoing phase III randomized controlled trial (EAGLE FM) to address the extent of dissection; however, the estimated completion date is not until 2030 [5]. In this era of shrinking indications for lymph node surgery in melanoma, it is important to consider whether ilioinguinal dissection adds value or whether it simply increases postoperative complications. Furthermore, it is important to consider how patients are selected for ilioinguinal dissection and whether the selection criteria in use are reasonable.

To this end, we sought to (1) describe practice patterns of groin dissection for melanoma, (2) investigate the outcomes of inguinal and ilioinguinal groin dissection, including both the short-term perioperative and long-term oncologic outcomes, and (3) understand surgeon preferences and perceptions on extent of surgery.

## 2. Materials and Methods

### 2.1. Study Design and Setting

A mixed-methods approach was undertaken using both a quantitative evaluation of surgical practice patterns and a qualitative approach to understanding surgeon preference and perceptions. Study approval was obtained from the institutional review boards of all institutions.

### 2.2. Surgical Practice Patterns

A retrospective review of three prospectively maintained databases (Synoptec, AB, provincial, population based datatabase) from: Saint John, NB, Canada; Sunnybrook Health Sciences Centre, Toronto, ON, Canada; and The Ottawa Hospital, Ottawa, ON, Canada. The review was performed to abstract relevant data. All patients who were diagnosed with cutaneous melanoma and underwent a groin dissection between June 2004 and May 2019 were included. Due to the significant differences in natural history and prognosis of mucosal melanomas [6], as well as their low representation within the study cohort, mucosal primaries were excluded. Unknown primaries were included.

Variables collected included patient demographics (age, gender, body mass index (BMI) and co-morbidities), primary disease pathology (Breslow thickness, ulceration, mitoses, lymphovascular invasion (LVI) and satellitosis) and method of diagnosis of inguinal disease (sentinel lymph node biopsy (SLNB), palpable, radiographic; initial presentation vs. recurrence). In addition, data were collected on procedure(s) performed, node dissection pathology (total nodes, positive nodes and extra-nodal extension), postoperative course (complications, length of stay (LOS), readmission and mortality), lymphedema, adjuvant therapy, recurrence, status at last follow-up and death. Lymphedema was defined as use of compression stockings beyond the immediate postoperative period or a referral to a lymphedema clinic. A primary chart review captured any variables not contained within the databases. Patients and analyses were stratified based on their presentation of disease as sentinel lymph node positive vs. clinically positive (palpable or radiologically evident) and extent of dissection (inguinal vs. ilioinguinal dissection). The majority of dissections were performed prior to the publication of MSLT-II in 2017, thus most patients in the SLNB group went on to have completion groin dissection routinely.

### 2.3. Statistical Methods

Descriptive statistics were used to present patient demographics and practice patterns (Table 1). Median and interquartile range (IQR) were reported for continuous variables; frequencies and proportions were reported for categorical variables. Missing data were not replaced or estimated. The Mann–Whitney U-Test was utilized to compare continuous values. Categorical variables were compared using Pearson’s chi-squared test or Fisher exact test where appropriate. Kaplan–Meier survival analysis was used to display overall, recurrence free survival and cancer-specific survival (OS, RFS and CSS, respectively). Factors affecting survival were identified using multivariate Cox regression models. A two-sided *p*-value < 0.05 was used for statistical significance. Statistics were performed using SAS v.9.4 (SAS Institute, Cary, NC, USA). OS and RFS were calculated from the date of surgery to date of death or first recurrence of any kind (either local or distant), respectively. Time was censored at the date of last follow up for patients who were still alive or free from recurrence.

### 2.4. Surgeon Preferences and Perceptions

A qualitative approach was employed to better understand provider approach to decision making on extent of groin dissection. Semi-structured interviews were conducted amongst operating surgeons from the three database institutions. Questions were designed to capture surgeon specific approaches to groin dissection in addition to perceived risks and benefits. The interview guide was developed by the first author (SK) and piloted with the senior author (MLQ). All interviews were carried out by one interviewer (SK) via telephone or in person, and audio-recorded and transcribed verbatim. Anonymized transcripts were analyzed for unique ideas and common themes to saturation, whereby no new ideas were generated.

## 3. Results

### 3.1. Quantitative Results

#### 3.1.1. Entire Cohort

A total of 347 patients were reviewed, with a median age of 57.1, of whom 56.8% were female with a median BMI of 26.2. Overall, 7.2% had a thin melanoma, 49.9% had an intermediate thickness and 32.6% had a thick melanoma, of which 41.2% of primaries were ulcerated. The primary was unknown in 26 patients (7.5%). Patient characteristics and outcomes of the entire cohort are summarized in Table 1. Most patients were diagnosed with groin metastases during their initial presentation (64.6%) via positive sentinel node biopsy, while 35.2% presented as a clinically palpable disease in the groin. The median postoperative LOS was 2 days (1–4 IQR), with a complication rate of 46.7%. The vast majority of complications (81.3%) were low grade (Clavien–Dindo grade I-II). There were no grade V complications (death). The overall rate of lymphedema was 42.9%. Adjuvant therapy was given to 53.3% of patients.

#### 3.1.2. Sentinel Node Positive Cohort

A total of 170 patients (49%) were diagnosed with lymph node metastases on primary surgical treatment with wide local excision (WLE) and SLNB. For the vast majority of these patients (96.5%), SLNB was performed as part of their initial treatment for melanoma. For six patients (3.5%), SLNB was performed after a local or in-transit recurrence of a previously resected melanoma. The patients in this cohort did not have evidence of lymph node disease on preoperative imaging. Of these 170 patients, 141 (82.9%) went on to receive an inguinal dissection and 29 (17.1%) received an ilioinguinal dissection. Patient characteristics and outcomes are summarized in Table 2. The patient and tumour characteristics were very similar between the inguinal and ilioinguinal dissection groups, with the only significant differences being a slightly older patient (median age 62 vs. 54, *p* = 0.007) with a higher rate of ulceration (72.4% vs. 41.8%, *p* = 0.006) and satellitosis (34.5% vs. 8.5%, *p* = 0.002) in the ilioinguinal dissection group. There was a slightly higher rate of minor complications (grade I–II) in the ilioinguinal dissection patients, but there was no difference in the rate of serious complications (grade III or higher) or length of stay (LOS). There was a higher rate of extra-nodal extension (ENE; 27.6% vs. 8.5%, *p* = 0.014), and a correspondingly higher rate of adjuvant radiotherapy (17.2% vs. 3.5%, *p* = 0.014), in the ilioinguinal dissection group. While there was no difference in overall recurrence between groups, there was a trend towards decreased isolated deep node recurrence in the ilioinguinal dissection group (0% vs. 7.8%, *p* = 0.073). There was no significant difference in mortality or CSS. On multivariate analysis, there was no significant effect of type of procedure on survival.

#### 3.1.3. Clinically Positive (Palpable Disease or Imaging Positive) Cohort

A total of 177 patients (51.0%) were diagnosed with lymph node metastases on clinical exam or imaging (clinical exam/palpable 151 (85.3%), imaging 26 (14.7%)). For 60 patients (33.9%), the lymph node metastases were identified at their initial diagnosis of melanoma. For 116 patients (65.5%), the lymph node metastases represented a regional recurrence in the groin, i.e., lymph node disease that presented after initial treatment in which sentinel lymph node biopsy was either omitted or performed with negative results. Out of the clinically positive cohort, 69 (39.0%) received an inguinal dissection and 108 (61.0%) received an ilioinguinal dissection. Patient characteristics and outcomes are summarized in Table 2. There were no significant differences in patient characteristics between groups, apart from a higher rate of imaging detected disease in the ilioinguinal dissection group (21.3% vs. 3.5%, *p* = 0.002). There were no significant differences in perioperative outcomes between groups, including LOS, complication rates and lymphedema rates. In patients receiving an ilioinguinal dissection, the rate of positive deep nodes was 42.6%. There was also no difference between groups in receipt of adjuvant therapy, recurrence rates or patterns. Isolated deep node recurrence occurred in 10.1% of patients undergoing an inguinal dissection and 3.6% patients undergoing an ilioinguinal dissection (*p* = 0.17). There were no significant differences between groups on mortality or survival, and on multivariate analysis and there was no significant effect of type of procedure on survival.

### 3.2. Qualitative Results

Semi-structured interviews were conducted via telephone from 2017–2018 with 10 out of 11 surgeons operating on the patients contained in this study (90.9%). Interview duration ranged from 4.39–15.07 min (mean 8.54 min). Thematic saturation was achieved.

#### 3.2.1. Guidelines or Papers Used by the Surgeons

Resources cited by the surgeons as the basis for their approach included published guidelines from varying centres, textbooks, MSLT trials, case series, retrospective studies, personal literature reviews and multidisciplinary expert opinion. There was no consensus or common resources cited between surgeons.

#### 3.2.2. Estimated Percentage of Surgeon’s Dissections That Contain Deep Component

In total, 40% of surgeons (4/10) included a deep dissection “most of the time” or ≥75%. The other surgeons (5/10) estimated between 10–50%, with one surgeon estimating 1%.

#### 3.2.3. Surgeon-Level Indications for Deep/Pelvic Node Dissection

The majority of surgeons (9/10) stated that radiographic evidence of deep nodal disease would prompt an ilioinguinal dissection, although one surgeon added a caveat that it would also need to be unresponsive to systemic therapy. The majority of surgeons (8/10) also cited clinically evident disease (palpable) as an indication for the more extensive dissection. Most surgeons (7/10) also felt that extensive disease in the superficial chain, as in multiple positive sentinel nodes or intraoperatively positive sentinel nodes would be an indication for ilioinguinal dissection. Two surgeons also cited lymphoscintigraphic evidence of drainage to a pelvic node.

#### 3.2.4. Perceived Benefits of Deep Dissection

Nearly all surgeons (9/10) felt that the primary benefit of a deep dissection was loco-regional disease control. In addition, approximately half thought a deep dissection offered a small chance of improved disease-free survival. Notably, one surgeon did not feel there was any benefit to adding a deep dissection.

#### 3.2.5. Perceived Risks of Deep Dissection

Overall, most surgeons felt that the morbidity added by a deep dissection was low in comparison to the superficial dissection alone. Risks cited included injury to structures within the surgical field such as bowel, bladder, ureter and vessels, increased risk of hematoma, the possibility of abdominal wall or peritoneal hernias and pelvic pain. One surgeon felt that it increased the risk of lymphedema, although this view was not echoed in other interviews.

#### 3.2.6. Type of Incision Used

The majority of surgeons (6/10) interviewed used a “T-incision”, comprised of two separate incisions: an oblique incision superior to the inguinal ligament and a vertical incision inferior to the ligament. The remainder of surgeons (4/10) used the classic “lazy S” incision.

#### 3.2.7. *A Priori* Probability of Deep Nodal Disease That Would Prompt a Deep Dissection

The majority of surgeons (6/10) stated that they would include a deep dissection if the probability of having positive nodes was ≥10%. The remaining four surgeons would perform a deep dissection if the probability of positive nodes was 20–30% or greater.

## 4. Discussion

This is the largest multi-centre study of patients undergoing surgery for groin metastases in Canada to date. Moreover, it is unique in using a mixed-methods approach to better understand surgeon beliefs and perceptions on the indications for extended dissection.

### 4.1. Patient Selection and Deep Positivity Rate

Our study showed that almost 40% of patients undergoing groin dissection for melanoma received an ilioinguinal dissection, the majority of whom (78.8%) presented with clinically evident disease (either palpable or evident on imaging). All surgeons interviewed reported using a selective approach to ilioinguinal dissections with the main indications for deep dissection being clinically evident disease and radiographic evidence of deep nodal disease. Notably absent from the interview results was any mention of the use of Cloquet’s node. In the literature, the deep node positivity rate ranges from 9.3% with microscopically identified disease (i.e., positive sentinel node biopsy) to 55% with macroscopically identified disease (i.e., clinically evident disease) [7,8,9,10,11,12].

The selective approach used by the surgeons in this study produced a deep nodal positivity rate of 40.9%. This rate is at the higher end of the range reported in the literature, which is appropriate given that surgeons were predominantly performing deep dissections on patients with macroscopic disease. In addition, the deep nodal positivity rate found in this study was well above the *a priori* threshold (that would prompt the inclusion of a deep dissection) stated by all interviewed surgeons. This suggests that the indications for ilioinguinal dissection currently in use are reasonable. As described above, radiographic evidence was a key indication for ilioinguinal dissection for nearly all surgeons. However, preoperative CT has been shown to have poor accuracy for pelvic node involvement, with a reported sensitivity of approximately 60% [13]. While PET/CT is quite accurate for superficial groin metastases (sensitivity 97%), it does not perform much better than CT for deep groin metastases with a sensitivity of 67% and a false negative rate of 33% [14]. This suggests that while radiologic studies may be helpful in selecting patients for ilioinguinal groin dissection, they should not be the exclusive determinant of the extent of surgery.

### 4.2. Perioperative Outcomes

The median length of stay was similar across groups and subgroups in this study. In addition, there was no difference in complication rates between any of the groups, with the exception of a small increase in minor complications (Clavien–Dindo grade I–II) in the SNLB-ilioinguinal dissection subgroup (vs. the SNLB-inguinal dissection subgroup). While grade I and II complications are typically both underestimated and underreported, presumably this would have occurred to a similar extent in both groups. Other studies have echoed these findings, showing no difference in complications with the addition of a deep dissection [7,12]. There was no significant difference in lymphedema between groups, either by mode of presentation (SLNB-positive vs. clinically positive) or by extent of dissection (inguinal vs. ilioinguinal dissection). This finding is rendered more noteworthy by the fact that significantly more patients undergoing an ilioinguinal dissection received adjuvant radiotherapy, a known risk factor for lymphedema [15]. Although limited, the published data directly comparing the rate of lymphedema between the inguinal and ilioinguinal cohorts have not shown a significant difference [7,12]. Nearly all surgeons surveyed felt that the inclusion of a deep dissection did not add significant morbidity and certainly, this impression is supported by our results.

### 4.3. Oncologic Outcomes

Approximately half of all patients experienced a recurrence after groin dissection, which was similar regardless of extent of dissection. Patients in the clinically positive group recurred more frequently, earlier and had more distant events than the patients in the SLNB-positive group. This pattern of recurrences is consistent with previously published studies [16,17], and likely reflects the biology of the primary disease, as the clinically positive group had significantly higher risk pathology and higher stage disease compared to the SLNB-positive group. Within both the clinically positive and SLNB-positive groups, there was no difference in recurrence frequency or pattern with extent of dissection, although there was a trend towards increased isolated deep node recurrence with inguinal dissection. Looking at all patients undergoing an ilioinguinal dissection vs. an inguinal dissection, the difference in isolated deep node recurrence was significant (2.9% vs. 8.6%, *p* = 0.0011, respectively). This finding suggests that utilizing ilioinguinal dissection may improve regional control. Regional control, however, is also impacted by the delivery of adjuvant therapy, and there were significantly more patients receiving an ilioinguinal dissection who also received adjuvant radiation. In addition, more patients receiving ilioinguinal dissection received adjuvant systemic therapy, which also reduces locoregional recurrence [18]. This makes it difficult to determine the true impact of surgery on regional control. Few studies have directly assessed the impact of ilioinguinal dissection on deep node recurrence alone. Van der Ploeg et al. looked at 169 patients undergoing groin dissection for palpable metastases and did not find a difference in the pelvic node recurrence rate [7]. In a study by Egger et al., the rate of deep node recurrence was 11% in the cohort receiving a superficial-only dissection and 5% in the cohort receiving a combined dissection. While this difference did not reach statistical significance, the authors themselves note that their sample size was small (only 34 combined dissections) and there may well be a trend towards increased pelvic node recurrences in the superficial only group [12].

Another factor known to influence locoregional control is the quality of surgery performed. For instance, quality indicators such as lymph node yield and completeness of mesorectal excision have been well established in colorectal cancer surgery [19]. Quality indicators are less well established for melanoma surgery and this was not an area that was actively investigated in this study. However, the vast majority of the procedures included in this study were performed in a tertiary centre by a high volume of melanoma surgeons. The relationship between high volumes and improved outcomes is well documented in other solid tumours and similarly, there is evidence to support this association in melanoma treatment as well [20,21].

**Figure 1:**
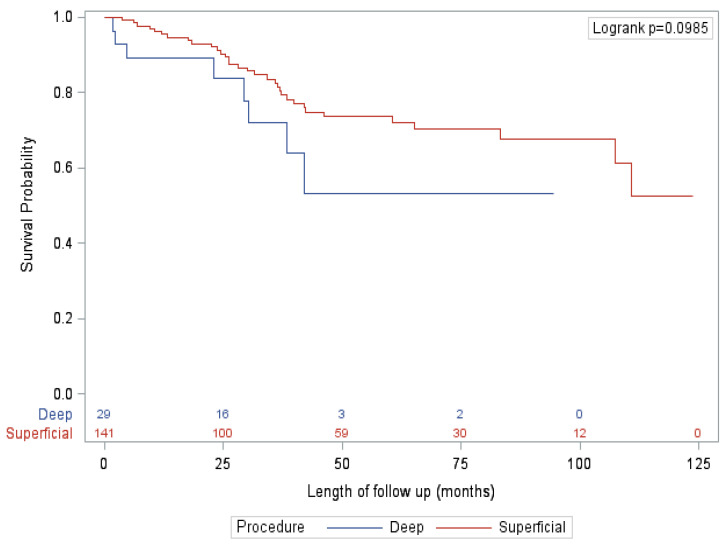
Overall survival—SLNB subgroup.

**Figure 2:**
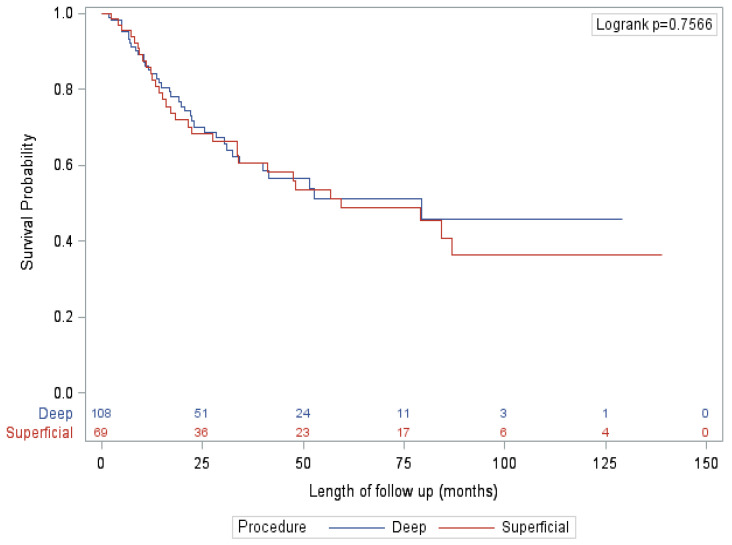
Overall survival—palpable/imaging subgroup.

After adjusting for known confounders, the only significant factor associated with survival on multivariate analysis was presented, with patients diagnosed by SLNB (vs. clinical exam/imaging) significantly associated with improved OS, RFS and CSS. Specifically, extent of procedure had no significant impact on survival (Figure 1 and Figure 2). This finding is mirrored in several other studies that did not show an association between extent of surgery and survival [16,22]. This finding is reflected in our qualitative results, where nearly all surgeons felt that the primary benefit of a deep dissection was regional control rather than improved survival. However, almost half of surgeons felt that there was a small possibility of improving overall survival as well. Looking closer at the data, this notion is also supported by evidence. Historically, the 10-year survival of patients with positive deep nodes is approximately 20% [9,17]. These numbers are derived from data that preceded effective systemic therapy, indicating that a reasonable proportion of patients with deep pelvic disease are cured by surgery alone. In this study, patients with positive deep nodes had a 5-year CSS, OS and RFS of 57.1%, 50% and 21.4%, respectively. For select patients, particularly given the lack of significant additional morbidity, outcomes may be improved by the addition of a deep dissection.

### 4.4. Limitations & Generalisability

This study cohort is comprised of patients who were selected to undergo groin dissection for a variety of reasons. Importantly, the majority of patients were treated prior to the publication of MSLT-II, therefore there are many SNLB positive patients who proceeded directly to groin dissection rather than ultrasound surveillance. This may have affected our results as these patients would typically have a lower burden of disease. However, more than half of the patients in this study presented with clinically positive disease. These patients would have proceeded directly to therapeutic lymph node dissection in the post-MSLT II era as well. Therefore, the data remains relevant and applicable to current melanoma patients. While multivariable analysis does control for known confounders, selection bias is impossible to remove entirely and other unmeasured variables may influence findings. Nonetheless, the qualitative component of the study strengthens our results by providing information that is generally unavailable retrospectively, namely preoperative rationale, method of patient selection and intent of surgery. It is one of the largest studies to date looking at this type of cohort and includes a broad sample of patients from multiple institutions across multiple regions. Thus, our conclusions are broadly generalizable to other patient populations. In addition, this study is one of the few to look at both perioperative and oncologic outcomes. This allows us to determine a true risk/benefit assessment of deep dissection, which is the most important assessment from a patient and provider perspective.

## 5. Conclusions

An ilioinguinal dissection is a safe and well-tolerated procedure, with no difference in length of stay or serious complications compared to an inguinal dissection. Importantly, there was no increase in lymphedema rates with an ilioinguinal dissection. There was also no difference in mortality or survival between groups, despite higher risk pathology in the ilioinguinal dissection group. Given that an ilioinguinal dissection is not associated with significant morbidity, it should continue to be used selectively in the treatment of melanoma metastases to the groin. Furthermore, the current indications used by the majority of melanoma surgeons today, namely palpable disease and radiologic findings, produce an acceptable rate of deep node positivity and should continue to be utilized going forward. Randomized data is needed to clarify the role that ilioinguinal dissection may have in regional control and patient survival.

## Figures and Tables

**Table 1:** Patient demographics and outcomes.

Variables	Category	Total (*N* = 347)
Age	Median (IQR)	57.1 (47.3–68.4)
Sex	F	197 (56.8%)
	M	150 (43.2%)
BMI	Median (IQR)	26.2 (23.4–30.1)
Comorbidities—CHF	Yes	23 (6.6%)
Comorbidities—COPD	Yes	7 (2%)
Comorbidities—CRF	Yes	5 (1.4%)
Comorbidities—CVA	Yes	5 (1.4%)
Comorbidities—DM	Yes	29 (8.4%)
Known primary	No	26 (7.5%)
	Yes	320 (92.2%)
Thickness	Thin (≤1 mm)	25 (7.2%)
	Intermediate (1.01–4 mm)	173 (49.9%)
	Thick (>4 mm)	113 (32.6%)
	Unknown	36 (10.4%)
Clark level	≤III	108 (31.1%)
	IV/V	239 (68.9%)
Ulceration	No	148 (42.7%)
	Yes	143 (41.2%)
	Unknown	56 (16.1%)
Mitoses	No	95 (27.4%)
	Yes	252 (72.6%)
LVI	No	221 (63.7%)
	Yes	45 (13%)
Satellites	No	230 (66.3%)
	Yes	37 (10.7%)
Mode of diagnosis	Imaging	26 (7.5%)
	Palpable	151 (43.5%)
	SLNB	170 (49%)
Timing of diagnosis	Initial presentation	224 (64.6%)
	Recurrence	122 (35.2%)
Systemic Metastases	No	336 (96.8%)
	Yes	9 (2.6%)
Extent of dissection	Superficial only	210 (60.5%)
	Combined	137 (39.5%)
LOS—Initial	Median (IQR)	2 (1–3)
LOS—Total *	Median (IQR)	2 (1–4)
Complications	No	185 (53.3%)
	Yes	162 (46.7%)
Complications—Grade ^†^	<III	282 (81.3%)
	III/IV	65 (18.7%)
Lymphedema	No	198 (57.1%)
	Yes	149 (42.9%)
Adjuvant therapy (any)	No	160 (46.1%)
	Yes	185 (53.3%)
Adjuvant therapy—type of therapy	None	161 (46.4%)
	Radiation	57 (16.4%)
	Interferon	76 (21.9%)
	Immunotherapy	18 (5.2%)
	Combination	23 (6.6%)
	Clinical trial	12 (3.5%)
Adjuvant radiation	No	290 (83.6%)
	Yes	57 (16.4%)

* Including readmission(s) length of stay. ^†^ Clavien–Dindo classification.

**Table 2:** Patient demographics and outcomes by presentation and procedure.

Variables	Category	Palpable/Imaging (*n* = 177)			SLNB (*n* = 170)		
		Deep (*N* = 108)	Superficial (*N* = 69)	*p*-Value	Deep (*N* = 29)	Superficial (*N* = 141)	*p*-Value
Age	Median (IQR)	59.4 (48.5–70)	63 (48.6–75.2)	0.2987	62 (53–73.4)	54 (45–62)	0.007
Sex	F	68 (63%)	37 (53.6%)	0.2173	13 (44.8%)	79 (56%)	0.2703
	M	40 (37%)	32 (46.4%)		16 (55.2%)	62 (44%)	
BMI	Median (IQR)	26.2 (23.3–29.7)	27.6 (24.4–31.6)	0.2165	28.3 (24–30.8)	25.6 (22.6–29.4)	0.1277
Comorbidities—CHF	1	7 (6.5%)	11 (15.9%)	0.0423	3 (10.3%)	2 (1.4%)	0.0355
Comorbidities—COPD	1	3 (2.8%)	2 (2.9%)	1	1 (3.4%)	1 (0.7%)	0.3129
Comorbidities—CRF	1	1 (0.9%)	4 (5.8%)	0.0765			0.1594
Comorbidities—CVA	1	2 (1.9%)	1 (1.4%)	1	0 (0%)	2 (1.4%)	1
Comorbidities—DM	1	10 (9.3%)	7 (10.1%)	0.8454	3 (10.3%)	9 (6.4%)	0.4325
Known primary	No	17 (15.7%)	9 (13%)	0.621			1
	Yes	91 (84.3%)	60 (87%)		29 (100%)	140 (99.3%)	
Thickness	Thin (≤1 mm)	13 (12%)	7 (10.1%)	0.9449	1 (3.4%)	4 (2.8%)	0.081
	Intermediate (1.01–4 mm)	40 (37%)	27 (39.1%)		13 (44.8%)	93 (66%)	
	Thick (>4 mm)	32 (29.6%)	22 (31.9%)		15 (51.7%)	44 (31.2%)	
Clark level	≤III	44 (40.7%)	26 (37.7%)	0.6847	6 (20.7%)	32 (22.7%)	0.8134
	IV/V	64 (59.3%)	43 (62.3%)		23 (79.3%)	109 (77.3%)	
Ulceration	No	42 (38.9%)	22 (31.9%)	0.5012	7 (24.1%)	77 (54.6%)	0.0061
	Yes	35 (32.4%)	28 (40.6%)		21 (72.4%)	59 (41.8%)	
Mitoses	No	38 (35.2%)	34 (49.3%)	0.0627	2 (6.9%)	21 (14.9%)	0.374
	Yes	70 (64.8%)	35 (50.7%)		27 (93.1%)	120 (85.1%)	
LVI	No	61 (56.5%)	35 (50.7%)	0.4383	20 (69%)	105 (74.5%)	0.4656
	Yes	8 (7.4%)	9 (13%)		7 (24.1%)	21 (14.9%)	
Satellites	No	57 (52.8%)	40 (58%)	0.7529	17 (58.6%)	116 (82.3%)	0.0019
	Yes	9 (8.3%)	6 (8.7%)		10 (34.5%)	12 (8.5%)	
Mode of diagnosis	Imaging	23 (21.3%)	3 (4.3%)	0.0019			
	Palpable	85 (78.7%)	66 (95.7%)				
	SLNB				29 (100%)	141 (100%)	0.0433
Timing of diagnosis	Initial presentation	38 (35.2%)	22 (31.9%)	0.8439	28 (96.6%)	136 (96.5%)	1
	Recurrence	69 (63.9%)	47 (68.1%)		1 (3.4%)	5 (3.5%)	
	Unknown	1 (0.9%)	0 (0%)				
Systemic Metastases	No	104 (96.3%)	64 (92.8%)	0.2645	27 (93.1%)	141 (100%)	0.0283
	Yes	3 (2.8%)	5 (7.2%)		1 (3.4%)	0 (0%)	
	Unknown	1 (0.9%)	0 (0%)		1 (3.4%)	0 (0%)	
LOS—Initial	Median (IQR)	2 (1–4)	2 (1–3)	0.7634	3 (1–4)	2 (1–3)	0.1342
LOS—Total	Median (IQR)	2 (1–4)	2 (1–4)	0.8466	3 (1–4)	2 (1–3)	0.0679
Complications	No	48 (44.4%)	38 (55.1%)	0.1677	12 (41.4%)	87 (61.7%)	0.0433
	Yes	60 (55.6%)	31 (44.9%)		17 (58.6%)	54 (38.3%)	
Complications—Grade	<III	86 (79.6%)	53 (76.8%)	0.6561	24 (82.8%)	119 (84.4%)	0.7847
	III/IV	22 (20.4%)	16 (23.2%)		5 (17.2%)	22 (15.6%)	
Lymphedema	No	54 (50%)	40 (58%)	0.3	17 (58.6%)	87 (61.7%)	0.7565
	Yes	54 (50%)	29 (42%)		12 (41.4%)	54 (38.3%)	
Total nodes harvested	Median (IQR)	16 (12–21)	9 (7–13)	<0.0001	16 (13–19)	8 (6–11)	<0.0001
Positive nodes	No	5 (4.6%)	10 (14.5%)	0.0216	13 (44.8%)	116 (82.3%)	<0.0001
	Yes	103 (95.4%)	59 (85.5%)		16 (55.2%)	25 (17.7%)	
Positive deep nodes	No	62 (57.4%)	69 (100%)	<0.0001	19 (65.5%)	141 (100%)	<0.0001
	Yes	46 (42.6%)	0 (0%)		10 (34.5%)	0 (0%)	
Extra-nodal extension	No	53 (49.1%)	32 (46.4%)	0.7079	17 (58.6%)	101 (71.6%)	0.0143
	Yes	46 (42.6%)	33 (47.8%)		8 (27.6%)	12 (8.5%)	
Adjuvant therapy (any)	No	45 (41.7%)	27 (39.1%)	0.7376	12 (41.4%)	76 (53.9%)	0.1594
	Yes	63 (58.3%)	42 (60.9%)		16 (55.2%)	64 (45.4%)	
Adjuvant therapy—type of therapy	No	44 (40.7%)	27 (39.1%)	0.8154	13 (44.8%)	77 (54.6%)	0.0008
	Radiation	31 (28.7%)	16 (23.2%)		5 (17.2%)	5 (3.5%)	
	Interferon	11 (10.2%)	11 (15.9%)		4 (13.8%)	50 (35.5%)	
	Immunotherapy	7 (6.5%)	5 (7.2%)		3 (10.3%)	3 (2.1%)	
	Combination	11 (10.2%)	6 (8.7%)		3 (10.3%)	3 (2.1%)	
	Clinical trial	4 (3.7%)	4 (5.8%)		1 (3.4%)	3 (2.1%)	
Adjuvant radiation	No	77 (71.3%)	53 (76.8%)	0.4177	24 (82.8%)	136 (96.5%)	0.014
	Yes	31 (28.7%)	16 (23.2%)		5 (17.2%)	5 (3.5%)	
Recurrence	No	41 (38%)	28 (40.6%)	0.1636	14 (48.3%)	84 (59.6%)	0.503
	Combined	13 (12%)	6 (8.7%)		5 (17.2%)	12 (8.5%)	
	Distant	39 (36.1%)	17 (24.6%)		4 (13.8%)	15 (10.6%)	
	Local	0 (0%)	1 (1.4%)		0 (0%)	3 (2.1%)	
	Regional	15 (13.9%)	17 (24.6%)		6 (20.7%)	27 (19.1%)	
Isolated deep node recurrence	No	63 (58.3%)	34 (49.3%)	0.1725	15 (51.7%)	46 (32.6%)	0.0728
	Yes	4 (3.7%)	7 (10.1%)		0 (0%)	11 (7.8%)	
	Unknown	41 (38%)	28 (40.6%)		14 (48.3%)	84 (59.6%)	
Time to recurrence	Median (IQR)	6.2 (3.6–13.7)	7 (3.4–18.7)	0.5512	18.4 (4.2–24.4)	15.5 (8.8–25.3)	0.6031
Follow up	Median (IQR)	22.2 (11.1–41.7)	32.4 (11–74)	0.1721	27.4 (8.2–38.6)	38.3 (22.4–70.2)	0.0034
Status at follow-up	Alive, disease	27 (25%)	11 (15.9%)	0.2171	11 (37.9%)	20 (14.2%)	0.0094
	Alive, no disease	46 (42.6%)	28 (40.6%)		12 (41.4%)	88 (62.4%)	
	Dead	35 (32.4%)	30 (43.5%)		6 (20.7%)	33 (23.4%)	
Mortality	No	70 (64.8%)	38 (55.1%)	0.1949	21 (72.4%)	108 (76.6%)	0.6316
	Yes	38 (35.2%)	31 (44.9%)		8 (27.6%)	33 (23.4%)	
Cancer specific	No	74 (68.5%)	43 (62.3%)	0.3954	23 (79.3%)	110 (78%)	0.8776
	Yes	34 (31.5%)	26 (37.7%)		6 (20.7%)	31 (22%)	

## Data Availability

The data presented in this study are available on request from the corresponding author. The data are not publicly available due to privacy and ethical constraints.
